# Changes in Communication after Transition from Paging to Collaborative Text Messaging

**DOI:** 10.1016/j.acepjo.2026.100482

**Published:** 2026-08-10

**Authors:** Deesha Sarma, Evan L. Leventhal, David T. Chiu, Bryan A. Stenson, Larry A. Nathanson

**Affiliations:** Department of Emergency Medicine, Beth Israel Deaconess Medical Center, Boston, Massachusetts, USA

**Keywords:** communication burden, workflow interruptions, secure chat messaging, emergency department, electronic health record

## Abstract

**Objectives:**

We report the impact on communication in a tertiary care emergency department that migrated from a homegrown electronic health record with tightly integrated unidirectional paging system to one with bidirectional secure chat messaging. The goal was to examine how this transition impacted communication frequency among emergency medicine resident physicians. We hypothesized that for each resident's shift, the ease of initiating electronic conversations would lead to an increase in the number of unique conversations (threads), as well as an increase in the total number of messages received.

**Methods:**

We conducted a retrospective observational study over two 6-month periods. The number of incoming pages residents received per shift in the preperiod was compared with the number of received secure chat messages as well as message threads per shift in the postperiod using nonparametric testing.

**Results:**

Residents received a median of 42 pages (IQR 27 to 62) per shift in the preperiod. They received a median of 107 secure chat messages (IQR 63 to 164) and participated in a median of 28 message threads per shift in the postperiod (IQR 18 to 41). The differences between each of these groups were statistically significant (Kruskal–Wallis *P* < .001).

**Conclusion:**

The transition to collaborative text messaging functionality doubled the number of messages received by residents per shift, but with fewer discrete message threads. The findings suggest secure chat may increase overall message volume but consolidate communication into longitudinal conversations. Future work will measure the impact of these messages on clinical productivity, safety, as well as optimization of communication cadence and prioritization.

**The Bottom Line**

Does switching from pagers to secure chat messaging change the nature of asynchronous communication in the emergency department? In this retrospective observational study at an academic emergency department that transitioned to a new electronic health record, resident physicians received more than double the number of secure chat messages (107 per shift) but were involved in significantly fewer chat threads (28 per shift) compared with pages (42 per shift) in the old system. This suggests that, whereas secure chat functionality facilitates an increased volume of messaging, this is consolidated over fewer threads and, thus, may mitigate workflow interruptions and cognitive load.

## 1 Introduction

### 1.1 Background

Communication among all members of the health care team, from physicians and nurses to consultants and ancillary staff, is an essential part of providing safe, comprehensive care of patients in the emergency department (ED). In large facilities where immediate face-to-face communication may not always be feasible, this often takes place in the form of text-based messaging, either through a paging system or secure online messaging integrated with the electronic health record (EHR). Regardless of whether communication takes place face-to-face or electronically, it often represents a cognitive interruption for the emergency physician.^1^

### 1.2 Importance

Over time, these patient care interruptions can increase stress and workload, and ultimately facilitate health care worker burnout.^2^ And although secure messaging has emerged as a dominant, HIPAA-compliant mode of communication, its impact on workflow and communication burden has not been extensively studied. One cross-sectional study highlighted the ease of sending messages over an EHR-integrated secure chat platform, and found that this resulted in short message response times and high messaging volume.^3^ Another single-center study reported improved satisfaction among health care providers with the implementation of a secure chat system in addition to the existing direct-paging system.^4^ However, although secure messaging may reduce the number of phone calls or pages, the volume of messages and expectation of prompt response times may shift, but not reduce, interruptions, and therefore not meaningfully impact workplace stress.^2^

### 1.3 Goals of This Investigation

Our ED migrated from a homegrown, web-based electronic health record platform to a new EHR in June of 2024, and in doing so moved from a unidirectional paging system to one that used bidirectional secure chat messaging. In the former system, all providers carried an assigned pager while on shift. Pages consisted of transmitted messages sent to the pager, including both automatic and personalized pages. These were tracked through the electronic paging system with timestamps. Personalized pages were sent through the EHR by selecting the recipient’s name in the patient chart; the chart listed all providers involved in the care of the patient, including the primary emergency physician team, nursing staff, and consulting services. Automatic pages included system-generated messages regarding bed assignments, electronic sign-out acceptance by admitting teams, and critical laboratory values.

After the transition, users accessed a chat function within the EHR that could be accessed either through the desktop or their smartphone. The chat could be linked to a specific patient and allowed users to participate in discussions with multiple other users. Chats could be viewed and joined by any user logged into the EHR during the encounter. This communication transition represented an opportunity to investigate the impact on providers, in particular, emergency medicine resident physicians. We hypothesized that for each resident physician's shift, the ease of initiating electronic conversations would lead to an increase in the number of unique conversations, as well as an increase in the total number of messages received.

## 2 Methods

We conducted a retrospective observational study at our urban, tertiary care academic medical center. The ED is staffed by resident physicians, under the supervision of attending physicians, who are the primary point of contact for other members of the health care team, including nurses, technicians, and consultants. Each shift in the ED is scheduled for 8 to 9 hours. There were two 6-month periods studied. The first period was July 1, 2023, through December 31, 2023. During this period, the primary electronic means of communication between care team members was through pagers. Pages could be sent automatically (eg, bed assignment notifications) or directly from the EHR. All pages received by emergency medicine resident physicians were compiled. The transition to the new EHR and secure chat system took place on June 1, 2024. After a 1-month run-in period, the second period studied was July 1, 2024 through December 31, 2024. After the EHR migration, messages were now sent through the EHR. A message thread involved 2 or more participants, and each line in the chat was counted as a single message. All message threads involving emergency medicine resident physicians were compiled. The number of pages received in the preperiod was compared with the number of participating message threads, as well as the number of individual secure chat messages received by residents per shift in the postperiod.

Significance testing was performed between these groups using the Kruskal–Wallis test, followed by post-hoc comparison using Dunn’s test with Bonferroni correction. The study was approved for exemption status from the Institutional Review Board.

## 3 Results

In the preperiod, emergency medicine resident physicians received a median of 42 pages per shift (IQR 27 to 62). In the postperiod, they received a median of 107 individual secure chat messages per shift (IQR 63 to 164). This difference was statistically significant (*P* < .01). In the postperiod, they were part of a median of 28 secure chat message threads per shift (IQR 18 to 41). Differences between pages, secure chat messages, and message threads were statistically significant using Kruskal–Wallis testing (H = 3402.3, *P* < .001). Post-hoc analysis with Dunn’s test with Bonferroni correction demonstrated significant pairwise differences between all groups (*P* < .001).

In the postperiod, 10% of message threads were initiated as automated notifications, and 17% of message threads were related to discussion with consulting services. In 73% of message threads, the resident physician was an active participant, defined as having sent at least 1 message in the chat.

In summary, after the EHR migration and implementation of secure chat messaging, there was a statistically significant increase in the number of individual messages received, yet significantly fewer message threads, compared with pages.

## 4 Limitations

Although this study begins to elucidate the communication differences that arise with a transition from a traditional paging system to online chat messaging, there were several limitations. Although the quantity of messages was calculated, the nature of this communication was not. In a page, the full message must be typed out within a set number of characters. But within a secure chat platform, a message can be split up across multiple lines. In addition, the bidirectional nature of the system could encourage general messages not specifically related to patient care, such as greetings, thanks, or words of acknowledgment like “okay” and “sure.” This could contribute to the increased number of individual messages received by resident physicians, which more than doubled from the pre- to postperiod. However, regardless of the substance of the message, the process of receiving a chat may represent a workflow interruption. Of note, there is a functionality built into the EHR that allows users to preview the chat message without navigating away from their current screen, which may help limit these interruptions.

## 5 Discussion

The transition from paging to an EHR-integrated secure chat messaging system altered the nature of asynchronous communication in the ED. Although the number of messages received by residents during a shift more than doubled, residents participated in fewer individual message threads. This finding suggests that the secure chat functionality consolidates communication into longitudinal conversations. Although residents are receiving a greater volume of messages, the batching of messages may reduce the cognitive load of task-switching compared with the multiple disconnected interruptions of the paging system.

Without information about the content of the messages, it is not possible to know how many of the pages in the preperiod were part of a message thread that could have been similarly grouped as part of one conversation. In the postperiod, 10% of messages were automated notifications, eg, bed assignments, or abnormal laboratory results. A categorization of preperiod pages, including the proportion of pages that were automated, would provide a better understanding of whether there was an increase in automated messages after the EHR transition that contributed to the increased number of messages in the postperiod. Similarly, a deeper analysis and more detailed categorization of the postperiod message threads might highlight conversations where the resident sends a message (and is, therefore, considered “active”) but is not a primary participant in the chat. An example would be if the resident were included in a group chat with the admitting hospitalist and a consulting service. In the preperiod, they would not have been included in the page. However, secure chat functionality easily allows for multiple participants in a message thread. This type of communication represents a new type of workflow interruption that did not exist in the preperiod.

Because asynchronous communication methods like pages and secure chats can lead to workflow interruptions, the finding that there were significantly more messages in the postperiod is important and merits further research to determine the impact of chat messaging on clinical productivity as well as stress and burnout. In terms of clinical productivity, prior research has found that the number of pages per hour received by residents did not decrease the number of patients seen per hour.^5^ However, there do not appear to be recent studies looking at the relationship between chat messages and resident physician productivity. Although it is possible that the fewer message threads in the postperiod improve physician productivity, further research is required to investigate this potential connection.

These opportunities for further research can also help inform optimization of communication cadence and prioritization to improve clinical workflows and prioritize patient safety.

## Author Contributions

EL, DC, BS, and LN conceptualized the study and designed the methodology. EL and LN collected data and conducted data analysis. DS drafted the manuscript. All authors revised the manuscript and approved the final version.

## Funding and Support

By *JACEP Open* policy, all authors are required to disclose any and all commercial, financial, and other relationships in any way related to the subject of this article as per ICMJE conflict of interest guidelines (see www.icmje.org). The authors have stated that no such relationships exist.

## Declaration of Generative AI and AI-Assisted Technologies in the Writing Process

During the preparation of this work, the authors used ChatGPT for proofreading and language editing. The authors reviewed and edited the output as needed and take full responsibility for the content of the published article.

## Conflict of Interest

All authors have affirmed they have no conflicts of interest to declare.
